# A reduction in ecological niche for *Trypanosoma cruzi*-infected triatomine bugs

**DOI:** 10.1186/s13071-019-3489-5

**Published:** 2019-05-16

**Authors:** Guiehdani Villalobos, Angela Nava-Bolaños, José A. De Fuentes-Vicente, Juan Luis Téllez-Rendón, Herón Huerta, Fernando Martínez-Hernández, Maya Rocha-Ortega, Ana E. Gutiérrez-Cabrera, Carlos N. Ibarra-Cerdeña, Alex Córdoba-Aguilar

**Affiliations:** 1 Hospital General “Dr. Manuel Gea González”, Secretaría de Salud, Mexico City, Mexico; 2 Departamento de Ecología Evolutiva, Instituto de Ecología, Universidad Nacional Autónoma de México, Mexico City, Mexico; 3 Instituto de Ciencias Biológicas, Universidad de Ciencias y Artes de Chiapas, Tuxtla Gutiérrez, Chiapas, Mexico; 4 Instituto de Diagnóstico y Referencia Epidemiológicos, Secretaría de Salud, Mexico City, Mexico; 5 CONACyT-Centro de Investigación sobre Enfermedades Infecciosas, Instituto Nacional de Salud Pública, Cuernavaca, Morelos Mexico; 6 Departamento de Ecología Humana, Centro de Investigación y Estudios Avanzados del IPN (Cinvestav), Unidad Mérida, 97310 Mérida, Yucatán Mexico

**Keywords:** Chagas disease, Niche, Niche centroids, Triatomine bugs, *Trypanosoma cruzi*, Co-evolution

## Abstract

**Background:**

Theory predicts that parasites can affect and thus drive their hosts’ niche. Testing this prediction is key, especially for vector-borne diseases including Chagas disease. Here, we examined the niche use of seven triatomine species that occur in Mexico, based on whether they are infected or not with *Trypanosoma cruzi*, the vectors and causative parasites of Chagas disease, respectively. Presence data for seven species of triatomines (*Triatoma barberi*, *T. dimidiata*, *T. longipennis*, *T. mazzottii*, *T. pallidipennis*, *T. phyllosoma* and *T. picturata*) were used and divided into populations infected and not infected by *T. cruzi*. Species distribution models were generated with Maxent 3.3.3k. Using distribution models, niche analysis tests of amplitude and distance to centroids were carried out for infected *vs* non-infected populations within species.

**Results:**

Infected populations of bugs of six out of the seven triatomine species showed a reduced ecological space compared to non-infected populations. In all but one case (*T. pallidipennis*), the niche used by infected populations was close to the niche centroid of its insect host.

**Conclusions:**

*Trypanosoma cruzi* may have selected for a restricted niche amplitude in triatomines, although we are unaware of the underlying reasons. Possibly the fact that *T. cruzi* infection bears a fitness cost for triatomines is what narrows the niche breadth of the insects. Our results imply that Chagas control programmes should consider whether bugs are infected in models of triatomine distribution.

**Electronic supplementary material:**

The online version of this article (10.1186/s13071-019-3489-5) contains supplementary material, which is available to authorized users.

## Background

Pathogens and hosts are co-evolving with each other. This interaction implies that the life history traits of either actor is evolutionarily and ecologically driven by the life history traits of the opposite actor [1]. At the phylogenetic level, for example, this has been widely evidenced by the close evolutionary radiations between pathogens and hosts in many different taxa [2–4].

Parasites can modulate their host’s niche in two ways [5]. First, parasites can alter the behavior and/or morphology of the host, thereby driving its niche specialization [6]. For example, for cases where parasites manipulate their hosts, infected and non-infected host individuals will show trophic divergence as they are likely to differ in spatial and/or resource use [6]. An illustrative study case is that of acanthocephalans that parasitize freshwater amphipods and whose effects induce behavioral changes by amphipods to facilitate parasite transmission to the definitive bird host [7, 8]. In this example, infected amphipod hosts make use of different food resources compared to non-infected amphipods leading to different infection-dependent trophic roles in freshwater ecosystems [9]. Secondly, by modulating a host’s micro-ecological changes, parasites can lead to a host’s niche restriction so that the latter cannot maximize their niche breadth [10]. A related example to this restriction applies to explain distribution of parasites with complex life histories (for example, a parasite with an intermediate and definitive host), and whose distribution is expected to be narrower than that of their hosts [11]. In this case, the requirement for multiple hosts renders parasites more susceptible to local extinction (e.g. if hosts in some areas are more resistant) compared to parasites that require only one host to complete their life-cycle. However, a niche restriction for parasite–host pairs [5, 10] has not been tested (a close related example is that of [12]). According to a niche restriction test, one would predict that infected hosts will have a more restricted ecological niche than non-infected hosts.

Members of the subfamily Triatominae (Hemiptera: Reduviidae) are vectors of *Trypanosoma cruzi*, the causal agent of Chagas disease. Chagas disease is endemic and highly important in Latin American countries as it causes around 12,000 deaths annually [13] and is difficult to control. *Trypanosoma cruzi* is a typical parasite with a complex life-cycle; it requires bugs to carry and transfer it to humans and other mammalian reservoirs where it is transmitted through blood-feeding and defecation on the reservoir’s skin [14]. Triatomines make use of different blood sources that include not only mammals, but birds and even reptiles [14]. However, *T. cruzi* can only replicate in mammals so these animals play a key role in the survival of the parasite [15, 16]. Different local and international programmes and resources have been devoted to controlling this disease, one of which is to look at the ecological niche of triatomines to predict ecological conditions where infections are more likely to occur [17–20]. However, estimates based on ecological niche constructions usually assume that all bug individuals are equally likely to bear the parasite. That is, studies of triatomine niche do not consider that infected and non-infected individuals may differ in niche characteristics and so researchers have carried out niche predictions using both types of individuals within the same group (e.g. [21, 22]). Despite this, several sources of information indicate this assumption is unlikely as bugs defend themselves against the parasite, which may result in individual variation in parasite infection: (i) even when high numbers of parasites can enter the bug, there is a massive mortality of the former [23] and immune responses by the latter [24], suggesting an active reaction of the bug against the parasite; (ii) there is variation in the number of bugs that become infected in natural conditions (e.g. 9–39% infection rates in *Triatoma phyllosoma* [25–27]; in *T. dimidiata*, differential *T. cruzi* prevalence (0–100%) and discrete typing unit (DTU) specificity for individual haplogroups has been reported [28] implying that some bugs are more susceptible to infection; and (iii) *T. cruzi* infection has a strong impact on life history traits [22, 29, 30] and leads to a reduction triatomine survival in a temperature-dependent manner [31–34], implying that such fitness costs explain resistance by bugs as indicated above. Given the above, modelling of triatomine ecological niche should consider whether bugs are infected or not as, according to the theoretical background, infected bugs may have a different niche use.

In this study, we tested if there are niche use differences between *T. cruzi*-infected and non-infected bug populations of seven triatomine species. In particular, we explored the environmental associations of infected *vs* non-infected bugs’ niche uses to evaluate whether niche centroids (i.e. the environmental space where populations are expected to be better adapted to their environments) were closer than expected given their niche amplitude. Additionally, we compared the niche size (i.e. range of the environmental conditions) of infected *vs* non-infected (per vector species). The analysis of the environmental space allowed us to understand the environmental determinants of the geographical distribution, i.e. whether infected vectors occurred preferentially in a restricted (and common) region of the ecological space that could restrict their distribution in the geographical space. We discuss our results in terms of both our understanding of factors driving distribution and niche usage in this triatomine–trypanosomatid system, as well as in the prevention of Chagas disease.

## Results

**Fig. 1:**
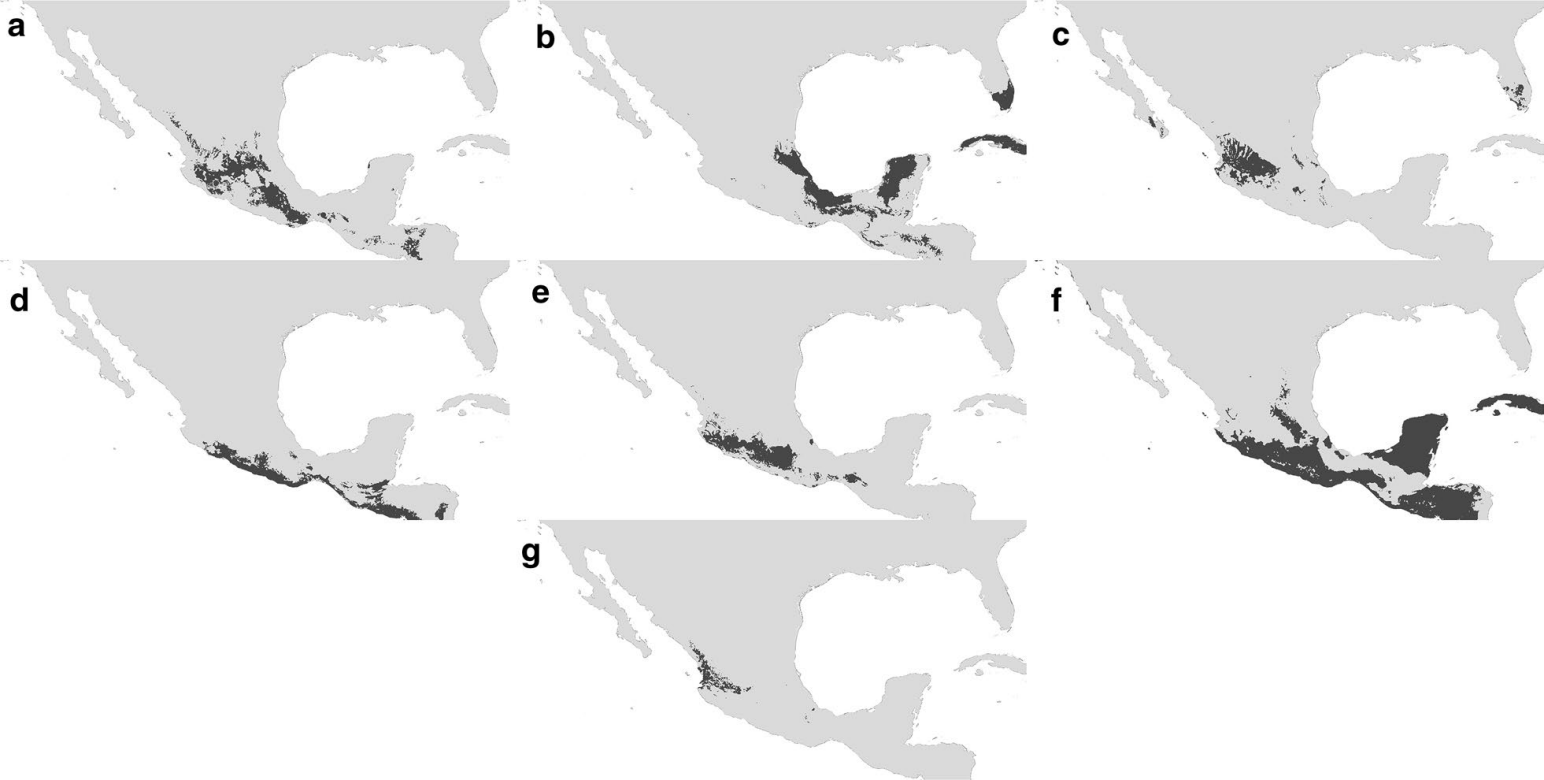
Potential triatomine distribution in Mexico and surrounding areas for *Triatoma barberi* (**a**), *T. dimidiata* (**b**), *T. longipennis* (**c**), *T. mazzottii* (**d**), *T. pallidipennis* (**e**), *T. phyllosoma* (**f**) and *T. picturata* (**g**)

**Fig. 2:**
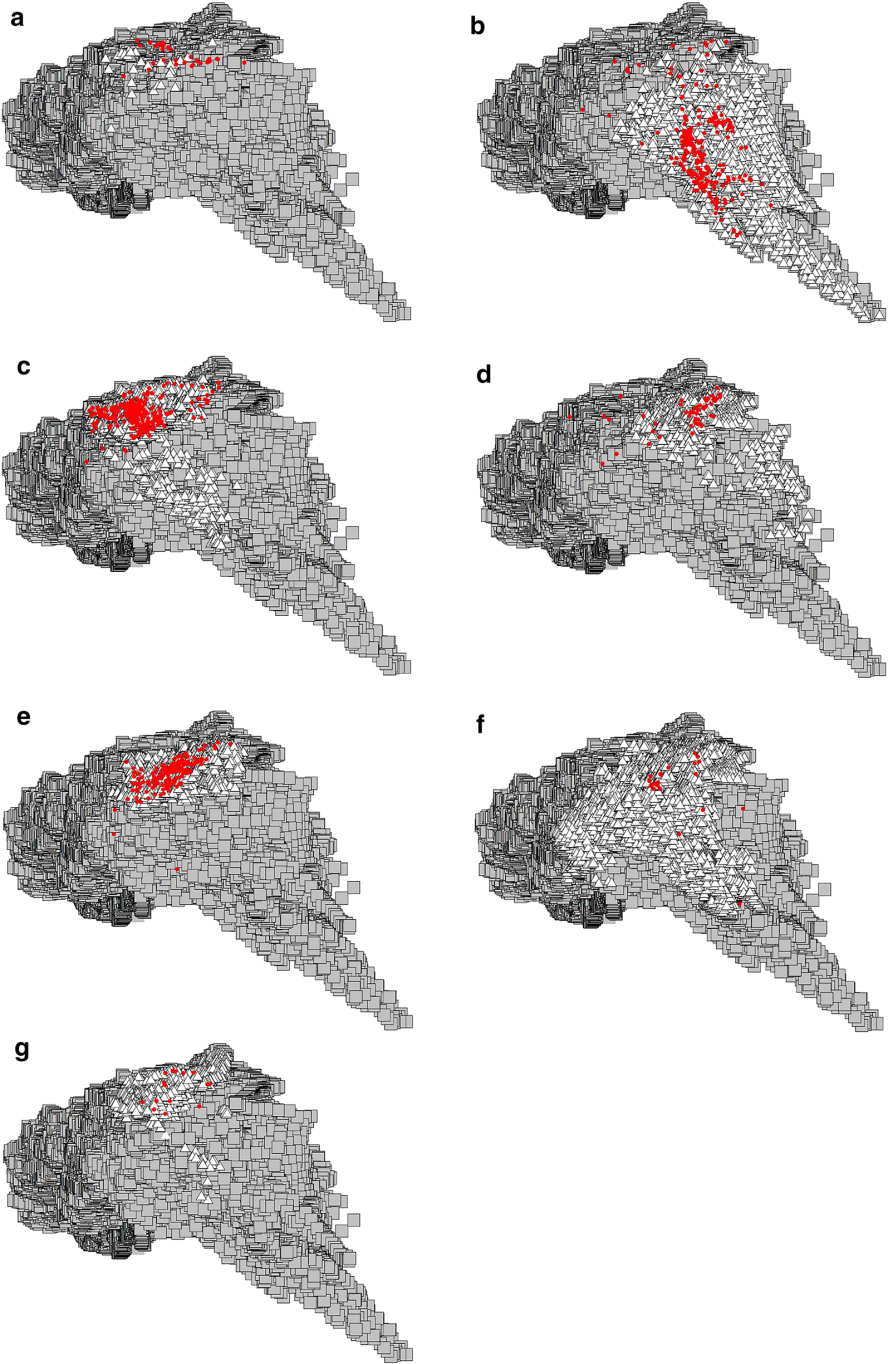
Environmental spaces for *Triatoma barberi* (**a**), *T. dimidiata* (**b**), *T. longipennis* (**c**), *T. mazzottii* (**d**), *T. pallidipennis* (**e**), *T. phyllosoma* (**f**) and *T. picturata* (**g**), based on the first two principal components (PCA) of the bioclimatic predictors. Gray quadrangles represent the universe of the environmental space, white triangles represent localities for each triatomine species, and the red circles are localities where infection for each triatomine species is present

**Fig. 3:**
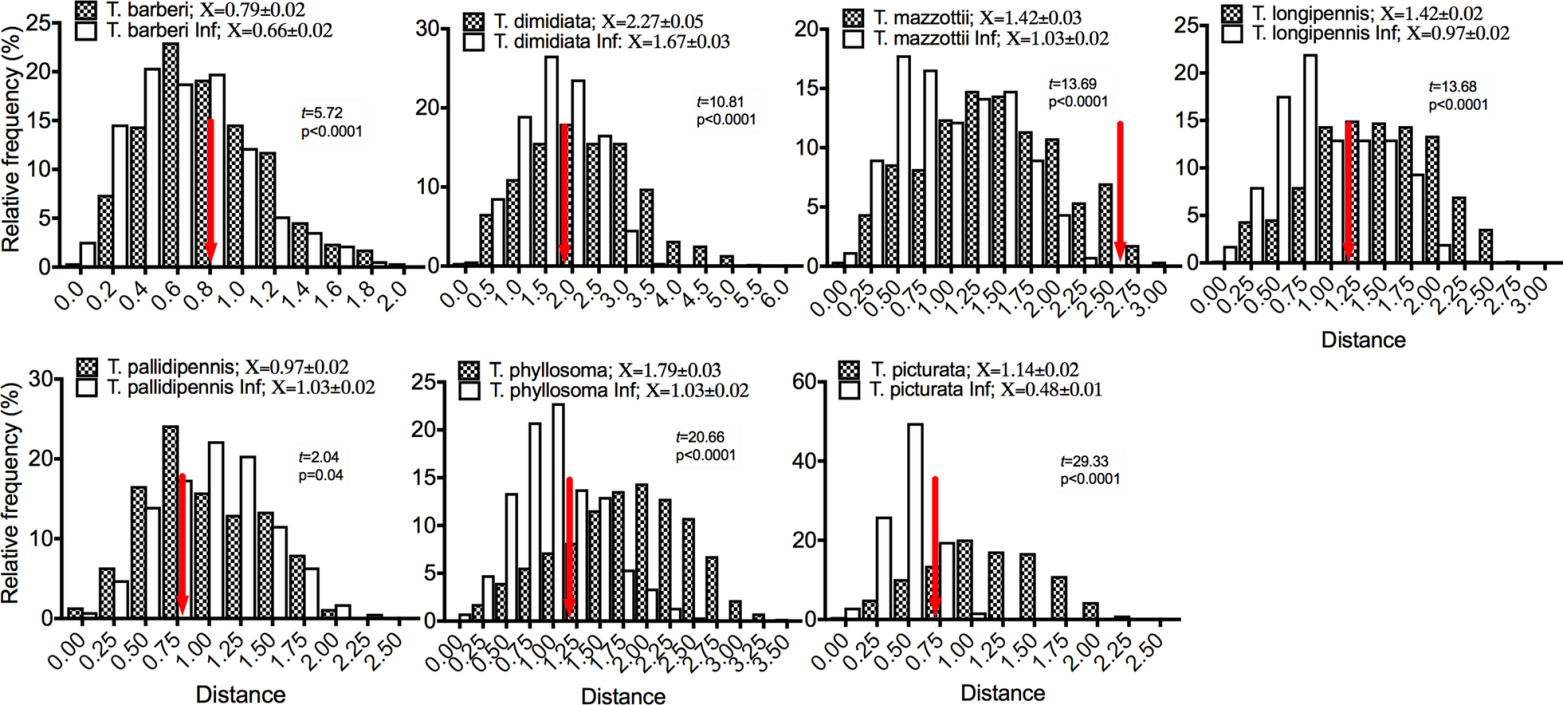
Niche amplitude (bars) and inter-centroid distance (red arrows) of triatomines and its infected populations. Student’s t-tests were used to determine whether the niche amplitude between these two groups was significantly different. Inter-centroid distance is contrasted with the mean distance between centroid and random points that characterize the species niche (mean and EE are shown for each species and its infected populations)

Models of current potential distribution showed that the suitability areas for all triatomine species were variable (Fig. 1). Based on the environmental PCA analysis (Fig. 2), it could be determined that infected populations use a reduced ecological space when compared with the ecological niche generated from non-infected populations (see Student’s t-test results in Fig. 3), except for the case of *T. pallidipennis* in which the reverse was observed (Fig. 3). By examining the average distance between the centroids of infected populations (21 combinations; mean distance = 1.6) compared to the average distance between infected and non-infected pairs (mean distance = 1.3), we could observe that no particular environmental envelope for infected populations exists (i.e. there is not a reduced parasite niche); furthermore, the average inter-centroid distance between all combinations of infected populations is not significantly different that the inter-centroid distance of all triatomines (one-tailed Student’s t-test, *t* = 1.12, *P* = 0.12; Table 1). In most cases (5 out of 7), the distances between niche centroids for infected and non-infected pairs were lower than the average distance of the non-infected niche centroids to their corresponding random points (Fig. 3). The two species whose inter-centroid distances were higher were *T. barberi* and *T. mazzottii*, the latter exceeding a standard deviation from the mean of centroid-random point distances.

**Table 1:** Inter-centroid distances between infected populations of all species (above the diagonal), triatomine species (below the diagonal) and between infected and non-infected pairs (diagonal, in boldface)

	*T. barberi*	*T. dimidiata*	*T. longipennis*	*T. mazzottii*	*T. pallidipennis*	*T. phyllosoma*	*T. picturata*
*T. barberi*	**0.80**	2.24	0.69	2.69	0.91	2.21	0.28
*T. dimidiata*	4.19	**1.87**	2.60	2.09	1.66	0.75	1.69
*T. longipennis*	0.92	3.28	**1.24**	3.28	0.97	2.04	0.92
*T. mazzottii*	3.15	1.90	2.42	**2.61**	0.74	2.11	0.43
*T. pallidipennis*	0.92	3.75	0.96	2.39	**0.81**	1.93	1.16
*T. phyllosoma*	1.75	2.45	0.83	1.82	1.50	**1.09**	2.22
*T. picturata*	0.97	3.50	0.75	2.22	0.27	1.23	**0.67**

## Discussion

As predicted, there were intra-specific differences in niche characteristics in *T. cruzi*-non-infected triatomines compared to *T. cruzi*-infected triatomines, with the latter showing a more restricted niche breadth. This relationship could not be explained by a restricted niche for *T. cruzi* as a whole, suggesting that parasite occurrence is more likely where triatomines are closer to their niche centroid. This difference may be a consequence of the cost exerted by parasites on triatomines, which can limit triatomines’ niche usage. As indicated before, a fitness cost for the host has been already shown in triatomines in terms of reduced survival in the face of infection [32]. This would select for defense mechanisms by the host which would explain, for example, the observed differences in triatomine’s immune ability against the parasite along altitude [23] and a reduction in *T. cruzi* population soon after infection [21]. Furthermore, since not all bug species show the same trend in niche use for infected and non-infected animals, we hypothesize that this pattern may be seen as the different aspects of a co-evolutionary arms race between *T. cruzi* and triatomines. In this regard, either some populations or even species may have a reduced fitness costs (e.g. the case of species whose infected and non-infected populations have similar niche “traits”). Towards testing these ideas, it would be interesting to compare fitness differences derived from *T. cruzi* infection in species where infected and non-infected populations do not differ in niche (e.g. *T. longipennis*) *vs* those population that do differ (e.g. *T. picturata*). We predict that fitness costs will be higher in species where niches of infected and non-infected populations differ in comparison to species where such niches for the same groups are the same.

A second important issue to explain our results is that there are factors other than fitness costs driving a niche use difference, such as, for example, preference for a specific food source [35–38]. Given the relatively large area extensions occupied by triatomines (both infected and non-infected populations, especially for *T. dimidiata*, *T. longipennis* and *T. phyllosoma*), other abiotic factors may also play a role. For example, Langford & Janovi [39] found that soil conditions [loam soils (comprising mainly sand, silt and clay), temperature (5–35 °C) and soil moisture (< 50%)] were key to determining infection by lungworms in frogs that occupy large distribution ranges. Although *T. cruzi* is not a free-living parasite like the infective stages of lungworms, it certainly depends on several species of mammals to complete its life-cycle [40]. Such plasticity in host use may imply that the parasite is unable to cope with all possible niche variations in terms of finding a host or a vector and reproducing within it. In general, host specificity determines how successful a parasite can be in invading or widening its habitat [41]. In fact, Chagas disease risk in humans is not associated with mammal host diversity [42] which provides support to the suggestion that *T. cruzi* is not fully able to reach as many hosts and vector species as possible. A third topic to understand niche differences and similarities in infected and non-infected populations is related to temperature [43]. In general, triatomines are heavily affected by temperature [44]. For example, hatching rates in infected *Rhodnius prolixus* triatomines are temperature-dependent [32]. A fourth topic that may determine parasite–triatomine distribution is that of anthropogenic factors. In this regard, studies of predictors of domestic areas have rarely included both actors simultaneously. For example, we know that predictors of triatomine occurrence include exterior (e.g. house age, upkeep, spatial location in the town [45]); public street lights [46]) and interior housing characteristics (number of inhabitants (over seven), overhead storage space, grain shed, cats, pigs and dogs [47]), altitude and mean annual precipitation [25]. As for the parasite, a likely predictor includes unplastered mud-stick houses [48]. When both actors have been found, the predictors for both were the accumulation of woodpiles [49], the periphery of rural villages [50] and presence of domestic mammals [51]. In summary, food preferences, temperature and anthropogenic factors are three candidate factors that may determine triatomine distribution and niche. In this regard, laboratory experiments should be combined with distribution to assess the niche properties to then locate which conditions better predict the geographical location of infected triatomines.

It should be noted that we did not control the type of *T. cruzi* discrete typing unit (DTU) in our analysis as we considered a broad category of infected animals. In Mexico, it is predicted that all six described DTUs (TCI-TCVI) exist [52]. The predicted distribution ranges overlap considerably for all six types within the country [52]. This implies that is likely that we have more than one DTU in our database. In fact, mixed infections by different *T. cruzi* genotypes have been found in *T. dimidiata* populations from the Yucatan Peninsula [53]. Unfortunately, the way infection was determined in our database (*via* direct microscopic observation), makes parasite genotyping impossible. Finally, a key conclusion is that our results should make people refine Chagas prevention programmes. Currently, for example, many of these programmes assume that all triatomines found in the wild are equally likely to harbor *T. cruzi* parasites which our results refute. These studies predict triatomine or mammal distributions with the aim of elucidating risk indices [16, 22, 54]. Thus, future studies should consider whether bugs bear *T. cruzi* parasites and, if possible, *T. cruzi* lineage to outline Chagas risks. In this regard, the present study can be used to guide the design strategies of triatomine control based on the geographical regions we depict according to our present models, and with this, optimize the allocation of resources.

## Conclusions

The present study provides evidence that *Trypanosoma cruzi* infection correlates with niche characteristics used by six triatomine bug species that occur in Mexico. Two implications of this are: (i) *T. cruzi* may restrict the triatomine ecological niche so that triatomines cannot maximize their ability to colonize their full ecological spectrum; and (ii) predictions of triatomine distribution and triatomine control strategies should take into account that *T. cruzi*-infected populations may be geographically located in more particular environments.

## Methods

### Data sources

We used data of presence of seven Triatominae species occurring within the Mexican territory: *T. barberi* (Guanajuato, Guerrero, Hidalgo, Jalisco, Mexico state, Michoacan, Morelos and Oaxaca states; Additional file 1: Table S1), *T. dimidiata* (Campeche, Chiapas, Colima, Guerrero, Hidalgo, Jalisco, Mexico state, Michoacan, Oaxaca, Puebla, Queretaro, Quintana Roo, Tabasco, Tamaulipas, Veracruz and Yucatan states; Additional file 1: Table S2), *T. longipennis* (Aguascalientes, Colima, Chihuahua, Guanajuato, Jalisco, Michoacan, Nayarit, Sinaloa and Zacatecas states; Additional file 1: Table S3), *T. mazzottii* (Guerrero and Oaxaca states; Additional file 1: Table S4), *T. pallidipennis* (Guanajuato, Guerrero, Jalisco, Mexico state, Michoacan, Morelos, Nayarit, Oaxaca and Puebla states; Additional file 1: Table S5), *T. phyllosoma* (Oaxaca and Guerrero states; Additional file 1: Table S6) and *Triatoma picturata* (Nayarit and Jalisco states; Additional file 1: Table S7). Data were gathered from diverse sources: (i) Entomology Laboratory, Institute of Epidemiological Diagnosis and Reference (InDRE/ Health Secretary; Mexico City); (ii) technicians and/or entomologists from the health services of each Mexican state and locality where there is a problem of domiciliary infestation. Note that the effort and periodicity of collection depended on each particular state and sanitary jurisdiction; (iii) Entomology Laboratory, National School of Biological Sciences, Instituto Politécnico Nacional (Mexico City) whose members collected specimens from intradomiciliary areas; and (iv) literature references that include collections from urbanized and non-urbanized areas (see Additional file 1: Table S8). Records used were those falling between 1999 and 2013 for all species as collection was more intense in this time window. When collected, samples were checked for the presence of *T. cruzi via* direct microscopic observation of fecal content at 40× magnification. Feces were obtained by abdominal pressure and diluted in phosphate buffered saline prior to microscope observation [55]. Although this method implies a detection error, it has been safely validated so the error is minor [56]. This validation is based on the fact that the high motility of the parasite makes it very conspicuous during microscopic surveillance [57].

### Environmental predictors

For bioclimatic variables, we used WorldClim v.1.4 (www.worldclim.org) data [58] at a cell size of 0.041666669. To establish a set of uncorrelated climatic variables, we intersected the variables with presence points, but removed some variables [BIO1, annual mean temperature; BIO2, mean diurnal range [mean of monthly (maximum temp − minimum temp)]; BIO3, isothermality [(BIO2/BIO7) × 100)]; BIO5, maximum temperature of warmest month; BIO6, minimum temperature of coldest month; BIO8, mean temperature of wettest quarter; BIO9, mean temperature of driest quarter; BIO10, mean temperature of warmest quarter; BIO12, annual precipitation; BIO13, precipitation of wettest month; BIO14, precipitation of driest month; BIO17, precipitation of driest quarter; BIO18, precipitation of warmest quarter; and BIO19, precipitation of coldest quarter] with an exploratory data analysis and a Pearson correlation analysis (i.e. any value > 0.7), a jack-knife contribution and biological knowledge of species. After this, the final data set included temperature seasonality (BIO4), temperature annual range (BIO7), mean temperature of warmest quarter (BIO11), precipitation seasonality (BIO15) and precipitation of coldest quarter (BIO16).

### Potential species distribution modeling

Species distribution models (SDM) were generated with Maxent 3.3.3k [59]. Among software that makes use of presence-only data, Maxent has proven to be reliable under different datum conditions and applications [60]. In fact, despite having faced a large theoretical and empirical discussion, it has become one of the most widespread programs for species distribution models [61]. Briefly, Maxent uses georeferenced data points where the species has been found together with environmental variables to estimate the distribution (geographical range) of a species by finding the distribution which has maximum entropy (i.e. is closest to geographically uniform) subject to constraints derived from their predicting variables [61]. A crucial component of model calibration is the delimitation of an area accessible for species dispersal [62], over a relevant time period, the M (mobility), in the BAM model [63]. As such, we assumed that the biogeographical provinces of Mexico [64] occupied by each triatomine species are the boundaries of the areas accessible to colonize. Therefore, all biogeographical regions containing at least one presence record of the presence database were used in combination to mask the environmental predictors for each species. To corroborate that the calibration area was appropriated (i.e. covering both suitable and unsuitable environmental combinations [65]), we visually inspected the distribution of species data points within the background area (i.e. the “M” region) in a biplot comprised by annual mean temperature and annual precipitation. Final models were constructed on a species-specific basis after testing predictive ability under different parameter settings that modify model complexity. For this task, we used the same data partition (random seed of 70% for training and 30% for testing), the regularization number (i.e. an inverse penalization parameter) by using 0.5, 1, 2, 3 and 4 and the model features (i.e. quadratic, linear, etc.), in order to have all possible combinations. Other parameters were kept to default (convergence = 10–5, maximum number of iterations = 500, background = 10,000). Duplicate records were removed. To generate an estimation of predictive accuracy, we ran bootstrap replicated type using 10 replicates. Finally, extrapolation and clamping were not allowed. All of this was done to find which combination of settings and variables generated the best outcomes, while minimizing the number of model parameters, as well as producing ‘closed’, bell-shaped response curves which guaranteed model calibration. The models were evaluated based on the highest area under the curve value (AUC), and lower AUC difference between training and testing outputs [60].

### Niche characterization

The values of each of the bioclimatic variables used in niche models were extracted to every pixel in the Mexican territory to produce a matrix where a principal component analysis was applied on the correlation matrix. Next, the eigenvalues for the two first principal components were aggregated, as they accounted for the 73.378% of the environmental variance. Using the potential distribution models of triatomine species (the final presence map) and the correspondent infected data points, a final matrix was produced that included eigenvalues for every pixel of the presence maps (distribution model of species *i* and infected data points of species *i*). These matrices were converted to shapefiles in ARCMap using the principal components environment as the space. Then, a minimum convex polygon was created to generate: (i) its geometric centroid and (ii) 500 random points; with that, a multi-distance matrix between the centroid and each of their correspondent 500 random points was created for each vector and infected populations. We observed whether the inter-centroid distance differed significantly (∞ = 0.05) of the average centroid-random points distances. Additionally, we tested whether these two groups (infected and non-infected populations), had the same range and position using Student’s t-test.

## Additional file

**Additional file 1: Table S1.** Geographical data for *Triatoma pallidipennnis*. **Table S2.** Geographical data for *Triatoma longipennis*. **Table S3.** Geographical data for *T. mazzottii*. **Table S4.** Geographical data for *Triatoma phyllosoma*. **Table S5.** Geographical data for *Triatoma picturata*. **Table S6.** Geographical data for *Triatoma dimidiata*. **Table S7.** Geographical data for *Triatoma barberi*. **Table S8.** References of geographical data for all species.

## Abbreviations

SDM: species distribution models
AUC: area under the curve
DTUs: discrete typing units
